# Gestational Diabetes Mellitus: Efficacy of Non-Pharmacological Interventions for Management and Prevention

**DOI:** 10.3390/healthcare13182261

**Published:** 2025-09-10

**Authors:** Naika Dubois, Isabelle Giroux

**Affiliations:** 1 Interdisciplinary School of Health Sciences, Faculty of Health Sciences, University of Ottawa, Ottawa, ON K1N6N5, Canada; 2 School of Nutrition Sciences, Faculty of Health Sciences, University of Ottawa, Ottawa, ON K1N6N5, Canada; 3 Institut du Savoir Montfort, Ottawa, ON K1K0M9, Canada

**Keywords:** gestational diabetes, management, prevention, intervention, pregnancy, health, lifestyle, nutrition, physical activity

## Abstract

**Background**: Gestational diabetes mellitus (GDM) is a type of diabetes diagnosed during pregnancy and its prevalence is on the rise around the world. GDM increases the risk of serious adverse health outcomes for the mother and child. Multiple types of non-pharmacological interventions have been developed for the management and prevention of GDM; however, there is a lack of clarity regarding their effectiveness. **Objective**: To summarize the evidence on the efficacy of non-pharmacological interventions in the management and prevention of GDM. **Methods**: For this integrative review, a comprehensive literature search was conducted in the databases MEDLINE, CINAHL, Embase, Scopus, and Web of Science. The methodology followed the integrative approach outlined by Whittemore and Knafl’s, and study quality was evaluated using the Mixed Methods Assessment Tool. **Results**: A total of 44 relevant studies were included. Key themes identified for GDM management were (1) nutrition therapy and physical activity, (2) social and psychological support, (3) digital tools, and (4) barriers and facilitators. For GDM prevention, themes were categorized into individual-level approaches, (5) lifestyle and supplements, and population-level approaches: (6) environmental factors, and (7) health in all policies. **Conclusions**: The growing prevalence of GDM is a major public health concern that requires the implementation of effective multi-level evidence-based strategies. Environmental, socioeconomic, and racial determinants of health have substantial impacts on GDM, highlighting the need to address the root causes of the illness. Further research is needed to support effective preventive and management measures beyond standard pharmacological treatment, so that evidence-based solutions can be applied to enhance and safeguard the health of current and future generations.

## 1. Introduction

Gestational diabetes mellitus (GDM) has become more prevalent globally over the past few decades and is anticipated to continue its upward trend [1,2]. This rise is driven by higher rates of obesity in women of reproductive age, delayed childbearing, and the implementation of revised diagnostic criteria [2]. GDM is characterized by any level of glucose intolerance that arises or is first detected during pregnancy [3]. The prevalence of GDM among pregnant women typically ranges from 3% to 20% [4]. This condition is transient and develops during gestation but subsides after delivery [5]. For women with a prior history of the illness, research indicates that the probability of having GDM in future pregnancies ranges from 30 to 80%, with prevalence rates differing according to risk factors [6]. Furthermore, the likelihood of developing type 2 diabetes (T2D) in the future is estimated to be more than seven times greater for women who have had GDM compared to those with normal glucose levels during pregnancy [7]. Risk factors for GDM include pre-pregnancy overweight or obesity, being 35 years or older, a personal or family history of diabetes, and belonging to high-risk ethnic groups such as South Asian, Hispanic/Latino, Indigenous, Black/African American, Pacific Islander, and Middle Eastern [4,8,9]. Fetal complications related to GDM include macrosomia, congenital abnormalities, growth retardation, premature birth, and stillbirth [10,11]. GDM also increases the risk of developing obesity and T2D for the offspring [12,13]. Overall, the healthcare cost for an individual with GDM is estimated to be at least 25% more than for a woman without the disease, indicating that the economic burden associated with this condition is substantial [14,15]. GDM has far-reaching implications on public health as it perpetuates the detrimental cycle of diabetes and obesity over successive generations [16,17]. This highlights the importance of effective interventions to prevent the development of GDM and facilitate its management to help reduce or delay its recurrence and complications.

Although various interventions have been developed to prevent and manage GDM beyond standard pharmacological treatments typically involving insulin therapy, there is a lack of clarity regarding their effectiveness. Therefore, this integrative review aims to summarize the evidence on the efficacy of these non-pharmacological interventions in the management and prevention of GDM.

## 2. Methods

This study used an integrative review methodology to systematically synthesize the existing body of literature. The integrative review approach is particularly valuable for exploring complex research topics, as it allows for the inclusion of studies with varying methodologies and thus enables the development of a comprehensive understanding of the phenomenon being studied [18,19]. The review was conducted in alignment with the five-step framework proposed by Whittemore and Knalf (2005) [19], which includes: (1) identification of the research problem, involving a clear articulation of the issue and objective of the review; (2) conduct of a structured literature search using a strategy that incorporate databases and additional sources as needed to ensure an exhaustive retrieval of relevant studies; (3) selection and appraisal of data, where studies are assessed according to methodological criteria appropriate to their design; (4) data analysis, which entail synthesizing results to identify central themes and patterns across different types of studies; and (5) presentation of the findings, which involve summarizing key results using tools such as tables to support comparison and interpretation across studies.

### 2.1. Search Strategy

This integrative review was conducted to address the research question: “What is known from the current literature regarding the efficacy of non-pharmacological interventions for the management and prevention of GDM?” A literature search was performed across the databases MEDLINE, CINAHL, Embase, PsycINFO, Scopus, and Web of Science, covering all available publications up to August 2024. In addition to searches in electronic databases, manual searches of reference lists from relevant sources were conducted to identify further material that might not have been captured through database queries. Inclusion criteria were peer-reviewed scientific articles available in full text and published in either English or French, consistent with the authors’ language proficiency. No restrictions were imposed on the year of publication or type of study design. Following the principles of integrative review methodology, which seeks to integrate diverse sources of evidence to achieve a comprehensive understanding of a phenomenon, both primary and review-level studies were included in the search. This encompassed systematic reviews and meta-analyses, which provide valuable synthesized evidence and highlight broader trends, thereby strengthening and complementing other types of evidence [20,21]. Exclusion criteria included commentaries, editorials, gray literature, unpublished works, and dissertations. Articles that did not contain information relevant to the research question were also excluded. To maximize the scope and depth of the search, a wide range of keywords and database-specific Medical Subject Headings (MeSH) were utilized in various combinations. The terms included “gestational diabetes”, “GDM”, “maternal diabetes”, “diabetes in pregnancy”, “intervention”, “program”, “approach”, “prevention”, “management”, “individual intervention”, “community based”, “population based”, “non-pharmacological”, “self-management”, “self-care”, “efficacy”, “efficiency”, and other relevant synonyms. Boolean operators “AND” and “OR” were employed to refine the search, and truncation and wildcard were applied where appropriate to broaden the yield. All search results were first processed through Covidence “www.covidence.org (accessed on 5 August 2024)” for duplicate removal and preliminary screening of titles and abstracts. Articles deemed potentially eligible were then exported to Zotero (version 6.0.36), where full-text screening was carried out in accordance with the inclusion and exclusion criteria.

### 2.2. Data Analysis and Quality Assessment

Data analysis was guided by thematic analysis using an inductive methodology based on the frameworks proposed by Popenoe et al. (2021) and Dwyer (2020) [22,23]. This strategy enables researchers to consider multiple perspectives, identify unanticipated themes, and minimize bias by grounding findings directly in the literature [22,23]. This is particularly valuable for integrative reviews, which synthesize evidence from diverse sources and require an approach capable of capturing complexity while revealing overarching patterns [22,23].

The quality of the studies included in the review was assessed collaboratively by both authors using the Mixed Methods Appraisal Tool (MMAT) version 2018. The MMAT was chosen for its ability to evaluate a variety of research designs including quantitative, qualitative, and mixed methods, making it especially suitable for integrative reviews [24]. This tool allows to assess study quality across five domains: clarity of research questions, suitability of data collection methods, adequacy of the study sample, rigor of analysis, and alignment of findings with data, with higher scores indicating stronger methodological quality [24]. This assessment tool is also recognized for its balanced combination of reliability, convenience, and methodological rigor [25].

## 3. Results

### 3.1. Characteristics of Included Studies

**Figure 1:**
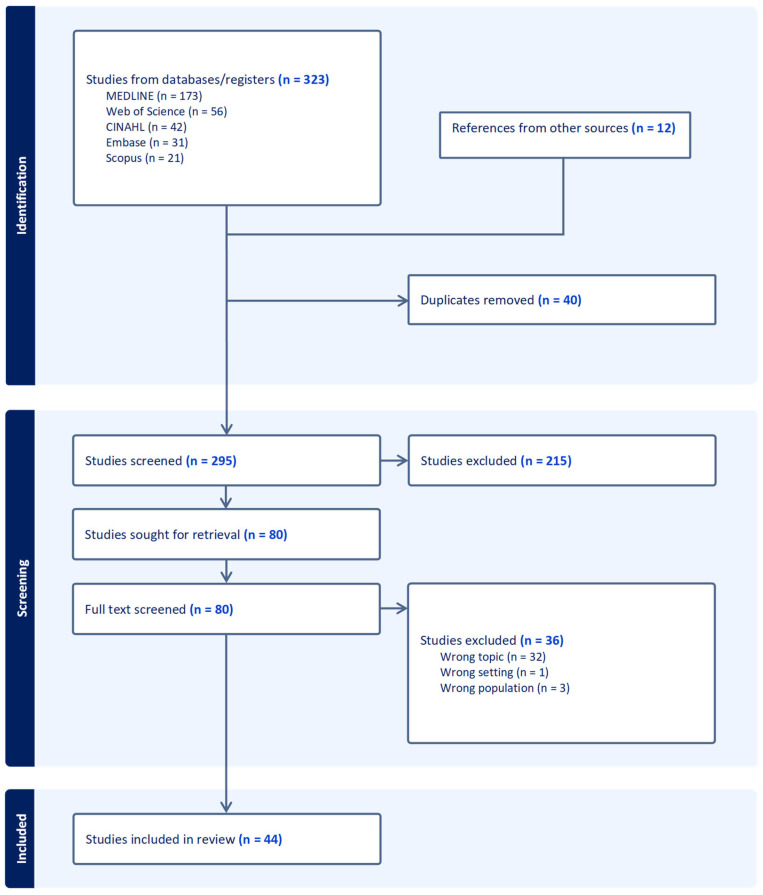
PRISMA flowchart.

From the literature search, a total of 323 studies were identified through database searches, with an additional 12 studies found via hand-searching relevant materials and bibliographic references. All retrieved articles were imported into Covidence for the removal of duplicates. Title and abstract screening were conducted within Covidence, after which the remaining 80 articles were exported to Zotero for full-text review. Based on the defined inclusion and exclusion criteria, 36 articles were removed, leaving 44 studies for inclusion in the review (see Figure 1). The characteristics of these included studies are detailed in Table 1. All retrieved studies were written in English; therefore, only English written articles were kept. Most included studies (39/44, 89%) have been published in the past 10 years (2014 to 2024). A large share of the selected articles are systematic reviews (22/44, 50%), including 15 meta-analyses. Regarding the quality assessment, all included studies demonstrated medium to high methodological quality. Among the 44 articles appraised, 25 (57%) obtained a score of 4/5, while 19 (43%) achieved the maximum score of 5/5. No study was rated below 4/5, reflecting the generally high quality of evidence (see Table 1).

### 3.2. Themes Identified from the Literature Search

The following themes were identified through inductive analysis and are discussed below:

Section 3.2.1. Interventions for GDM management beyond standard pharmacological treatment:
  - Nutrition therapy and physical activity;
  - Social and psychological support;
  - Digital tools;
  - Barriers and facilitators.

Section 3.2.2. Interventions for GDM prevention:
  Individual-level approaches:
    - Lifestyle and supplements.
  Population-level approaches:
    - Environmental factors;
    - Health in All Policies (HiAP).

#### 3.2.1. Interventions for GDM Management Beyond Standard Pharmacological Treatment

##### Nutrition Therapy and Physical Activity

Dietary recommendations for women with GDM appear to play a key role in controlling maternal gestational weight gain (GWG) and enhancing blood glucose management, resulting in better health outcomes for the mother and baby. Physical activity during pregnancy seems to promote blood glucose control and improve delivery outcomes for women diagnosed with GDM.

Evidence from systematic reviews suggested that dietary interventions during pregnancy can significantly reduce GWG and lower the risk of GDM, as well as lower the likelihood of cesarean sections, preeclampsia, and preterm birth [53,61,63]. Intensive dietary counseling combined with a low-glycemic index (LGI) diet, particularly when culturally tailored, has been shown to improve blood glucose levels and reduce pregnancy complications among Hispanic women with GDM [32]. According to findings from meta-analyses, dietary and lifestyle modifications helped reduce GWG without adversely impacting fetal growth, with dietary interventions demonstrating the greatest effect compared to physical activity or combined approaches [63]. Adopting a LGI diet and engaging in regular physical activity has also been found to reduce the risk of macrosomia [53]. Personalized nutrition therapy appeared most effective for optimizing blood glucose management, although general nutrition education was still beneficial for women with GDM [43]. Improved glycemic control has been associated with LGI and fiber-enriched diets among Chinese women with GDM [65]. According to meta-analyses data, physical activity during pregnancy was found to have a small protective effect against the onset of GDM but may assist in managing fasting and postprandial blood glucose levels, as well as hemoglobin A1c, in women already diagnosed with the condition [49,57]. Additionally, customized exercise regimens, particularly in the second and third trimesters, combined with dietary guidance, have been shown to improve blood glucose control, self-management, and delivery outcomes in women with GDM according to cross-sectional data [69].

##### Social and Psychological Support

Evidence indicates that women with GDM often experience depression, which may hinder effective disease management; however, psychological and social support interventions have been shown to improve health outcomes. 

Pregnant women with GDM were 3.79 times (OR = 3.79, 95% CI: 1.07–13.45, *p* = 0.04) more likely to have a history of depression than women without GDM, even after controlling for age, income, marital status, body mass index (BMI), and gravida, according to cross-sectional data [29]. Results from a prospective study indicated that women who lacked adequate social support during pregnancy reported higher rates of depression and a diminished quality of life [36]. Integrating comprehensive psychological support services in conjunction with treatment may enhance pregnant women’s ability to manage GDM more effectively [45]. An experimental study revealed that women with GDM reported experiencing less stress and enhanced control of their blood glucose levels after receiving mindfulness counseling during their pregnancy [68]. Moreover, improvement in GDM self-management as well as a decrease in GWG and macrosomia in infants was observed in a randomized controlled trial (RCT) where both the mother and her partner were involved in learning about GDM and its management [38]. Cross-sectional research further highlighted a strong positive correlation between social capital and self-efficacy in women with GDM [41].

##### Digital Tools

Mobile health (mHealth) and other digital resources seem beneficial in assisting and facilitating behavior modification and blood glucose level management for women with GDM.

Findings from a systematic review highlighted that digital tools were useful for women diagnosed with GDM by supporting them to adhere to their treatment, engaging in physical activities, and making healthier food choices, however research remains scarce [26]. A meta-analysis further indicated that the addition of mHealth therapies were more effective than standard care for patients with GDM and led to positive outcomes for mothers and infants [66]. Users of mHealth technology among GDM patients reported benefiting from motivational tools (goal setting, risk awareness, and problem solving) which helped them with behavior change and glycemic control according to a qualitative meta-synthesis [52]. Peer support via mHealth was also appreciated, such as being part of a network of women navigating similar challenges, allowing them to encourage one another throughout the process of GDM management and lifestyle modification [52]. However, participants also expressed that meaningful engagement with healthcare professionals remained a crucial component of effective support [52].

##### Barriers and Facilitators

For pregnant women with GDM, barriers to management often include negative psychosocial effects of the disease, difficulties dealing with the dietary and medical challenges involved, and adapting it to their unique family circumstances. Facilitating factors encompass social support, and the provision of culturally sensitive recommendations tailored to their health literacy and socioeconomic level.

Evidence from a scoping review highlighted that key facilitators of effective GDM management included support from family, friends, and broader social networks [28]. Conversely, common barriers involved inadequate knowledge, financial constraints, low motivation to adopt lifestyle changes, the high cost of healthy foods, and cultural or religious food practices [28]. Barriers to self-management have also been found to include mental strain, social isolation, stigma, and other adverse social and psychological effects associated with GDM [55]. Additional challenges included competing work and family responsibilities including childcare, as well as limited social capital [28]. A systematic review found that pregnant individuals with GDM who had limited knowledge of the condition exhibited higher blood glucose levels and demonstrated poorer self-care attitudes compared to those with a better understanding of GDM, its associated health risks, and effective management strategies [40]. Migrant women with low income and low literacy level were reported to struggle particularly with understanding and following nutritional and medical recommendations for the self-management of GDM, according to findings from qualitative studies and systematic reviews [30,31,32,43,44]. Furthermore, findings from a systematic review revealed that ethnic minority patients were more likely to trust healthcare providers when care was delivered in their preferred language, either directly or via interpreters, and adapted to their health literacy and socioeconomic level [48]. Interventions delivered in culturally familiar and accessible locations helped foster a sense of safety and comfort, thereby enhancing engagement, and served as facilitating factors [48]. Moreover, evidence indicated that women often struggle to prioritize their own dietary needs when these conflict with the interests of their families [64]. Results from a systematic review suggested that barriers to self-management and self-efficacy among women with GDM include a lack of individualized care, limited treatment options, inadequate follow-up options with their healthcare providers, and a feeling of abandonment after childbirth, which can negatively impact the adoption of lasting lifestyle changes [46]. Among facilitating factors, GDM mothers were more likely to be open to interventions when they understood they were making a positive difference for their baby [31,32].

#### 3.2.2. Interventions for GDM Prevention

##### Individual-Level Approaches

1. Lifestyle and Supplements

A growing body of evidence supports the use of both lifestyle modifications and targeted supplements, such as probiotics, myo-inositol and vitamin D, in reducing the risk of GDM, although findings remain inconsistent and at times contradictory. While no single strategy has proven universally effective, interventions that are personalized, initiated early, and supported by healthcare professionals or peer groups seem to yield the best outcomes.

Several recent meta-analyses have examined the impact of physical activity, dietary interventions, and supplements on GDM prevention. Studies reported that combined diet and physical activity interventions, as well as inositol and vitamin D supplementation, significantly reduced GDM incidence in high-risk women identified early in pregnancy [54]. Subgroup analyses suggested that lifestyle interventions were most effective among women with multiple GDM risk factors, while inositol supplementation appeared particularly beneficial for women with overweight or obesity [54]. Likewise, interventions involving lifestyle modification and myo-inositol were found to reduce GDM risk, especially when initiated preconception or during the early first trimester [51]. These strategies demonstrated greater efficacy in women without polycystic ovary syndrome (PCOS) and in those without a prior history of GDM [51]. Results from another recent meta-analysis found a decrease in the incidence of GDM when dietary, physical activity, or diet combined with physical activity therapies were compared to control measures [59]. In contrast, other meta-analytic findings indicated that while interventions targeting a single behavior, either diet or physical activity, can effectively reduce the risk of GDM, combining both into a comprehensive lifestyle intervention unexpectedly did not yield the same benefit [27]. On the other hand, physical activity and probiotic supplementation were found to significantly reduce GDM risk compared to placebo, whereas dietary changes, combined dietary and physical activity interventions, and inositol supplementation did not show significant effects, according to other meta-analytic data [62]. Other evidence suggests that physical activity-only therapies reduced the likelihood of developing GDM compared to no exercise at all, and exercise alone was more effective than programs combining it with other therapies [34]. Focused intervention with high-risk women utilizing a combination of dietary changes and exercises was found effective at reducing GDM risk, according to meta-analyses [39,51,54,58]. However, evidence from another meta-analysis showed that preventing GDM in overweight and obese women was not significantly helped by diet, physical activity or a combination of diet and exercise, even though those interventions were associated with lower GWG [67]. Pregnancy-related GDM risk appeared to be reduced in those who followed healthy eating habits before pregnancy such as the Mediterranean diet, according to a systematic review [35]. However, dietary interventions implemented during pregnancy were found to be less effective to prevent the development of GDM, particularly in high-risk groups [35]. Lastly, findings from a meta-analysis suggested that physical activity interventions carried out in healthcare facilities or in group-setting with supervision and feedback from professionals were found to be more effective at preventing GDM than those in community or individual settings [59].

##### Population-Level Approaches

1. Environmental Factors

The way urban spaces are designed can influence lifestyle habits, which in turn can promote physical and mental well-being and reduce the prevalence of certain diseases. Green space exposure during pregnancy is linked to better maternal glucose regulation, while low access to green space is associated with increased risks of GDM and other pregnancy complications, including preeclampsia, preterm birth, and depression. 

Findings from a prospective birth cohort study (N = 6807) indicated that greater access to green space was significantly associated with reduced maternal blood glucose levels and a lower risk of developing impaired glucose tolerance and GDM [50]. Similarly, a birth cohort study (N = 5814) reported that higher residential exposure to greenery was linked to lower levels of the hyperglycemic biomarker hemoglobin (Hb) A1c during mid-to-late pregnancy in women diagnosed with GDM [42]. A retrospective birth cohort study (N = 238,922) found that women living in areas with the lowest levels of green space faced the highest risks of developing GDM and mental health disorders [56]. Additionally, pregnant women with limited or poor access to green space within walking distance were more likely to experience preeclampsia, preterm birth, or depression [56]. Furthermore, in a prospective cohort study (N = 9155), researchers found that pregnant women residing in food deserts or less walkable neighborhoods had higher odds of developing GDM, after adjusting for known covariates [37].

2. Health in All Policies

To effectively address the growing burden of GDM, it is essential to acknowledge that health is influenced by factors beyond the healthcare system. The HiAP approach, which emphasizes collaboration across sectors to address the determinants of health, promotes comprehensive and preventive strategies addressing the root causes of diseases to improve population health and well-being.

Research highlights the potential of cross-sectoral collaboration in tackling persistent societal challenges such as perinatal health inequities [33]. These disparities arise from a complex interplay of social determinants, which requires coordinated action among professionals from local governments, healthcare, social services, and public health sectors [33]. The HiAP framework supports this by recognizing that health is deeply connected to social, cultural, environmental, and economic factors, and therefore requires cooperation across multiple sectors [33,60]. Active community engagement is also a key component of multisectoral strategies, particularly in addressing social determinants of health [47]. Consistent involvement of diverse sectors, agencies, and civil society can help co-develop culturally appropriate and effective interventions, while also fostering stronger communication and trust between communities and service providers [47]. Additionally, evidence indicates that achieving the HiAP agenda requires strategic leadership, strong data systems, sufficient financial investment, and well-coordinated intersectoral efforts [60].

## 4. Discussion

### 4.1. Discussion of Main Findings

The purpose of this integrative review was to summarize the evidence on the efficacy of non-pharmacological interventions in the management and prevention of GDM.

#### 4.1.1. Interventions for GDM Management Beyond Standard Pharmacological Treatment

##### Nutrition Therapy and Physical Activity

In terms of GDM management, which aims to control blood glucose levels within appropriate targets during pregnancy, the first line of treatment typically consists of dietary modifications and increased physical activity, along with regular blood glucose self-monitoring [4]. Insulin therapy is usually introduced only if blood glucose levels remain elevated despite these lifestyle modifications [4]. Findings from this review indicate that nutritional therapy can effectively prevent excess maternal gestational weight gain and improve glycemic control, leading to better outcomes for both mother and baby [32,43,53,61,63,65]. In addition, pregnant women with GDM who exercise regularly may have enhanced control of their blood glucose levels and safer delivery [49,57,69]. Research suggests that addressing systemic obstacles at the organizational level, such as offering childcare services, proved successful in facilitating increased physical activity among women with GDM [70].

##### Psychological Support

A growing body of evidence indicates that individuals diagnosed with GDM are particularly vulnerable to developing depression, which can negatively affect their overall treatment of the condition and blood glucose levels [29,71,72,73,74,75,76,77]. Research highlights that women with GDM have reported encountering stigma, often in the form of negative stereotypes that overlap with those associated with obesity and T2D [78,79,80,81]. However, GDM is uniquely perceived as a deviation from a normal pregnancy and is closely tied to societal expectations about the concept of being a “good mother” [79,80,81]. The stigma surrounding GDM is often amplified by these cultural expectations of ideal motherhood [79,80,81]. In many cultures, women are expected to put the well-being of the whole family before their own needs and to be responsible for domestic tasks and childcare, which can create major barriers to self-care and GDM management [82,83,84,85,86,87]. This often leads to internalized stigma, including guilt, self-blame, shame, and anxiety, and may negatively influence both physical and mental health, potentially leading to psychological distress and self-isolation [78,79,80,81]. It can also contribute to maladaptive coping behaviors, including avoidance of blood glucose monitoring, and low engagement with healthcare services [78,79,81,88,89,90]. Preexisting mental health issues can further complicate GDM self-management, which is concerning given the increased prevalence of past depression among this population [36,89,90]. Additionally, women affected by both depression and GDM tend to experience worse perinatal outcomes compared to those diagnosed only with GDM, according to findings from a cohort study in the United States, (N = 170,572) [75]. Evidence from a meta-analysis reported that GDM also contributes to an elevated risk of postpartum depression, with relative risk of 1.59 (95% CI: 1.22–2.07, *p* = 0.001) [71]. These findings underscore the need for routine psychological screening and individualized interventions to address GDM-related mental health challenges through specialized care.

##### Social Support

Findings from this review suggest that peer support is effective in helping individuals with GDM self-management and depressive symptoms [38,41,45]. In addition, social support networks have been found to influence not only maternal emotional well-being and quality of life, but also health behaviors and lifestyle choices during pregnancy, including dietary habits [36]. The size and quality of one’s social network were closely linked to health outcomes, as strong relationships provide emotional support that fosters better mental well-being and more effective decision-making in GDM self-management [41]. Low social support in pregnant women has been associated with weakened psychosocial resources, including diminished social integration and stability, which often translates into inadequate assistance during their pregnancy [36]. Disease management of GDM improved among women when their partners were actively engaged, offering both emotional encouragement and practical assistance [38]. Peers can also provide guidance on accessing affordable, nutritious food options and motivate one another to participate in group exercise activities within a safe environment [91,92]. Additionally, strengthening social capital among individuals with diabetes was found to improve blood glucose control, as those with cohesive family relationships, robust social networks, and frequent communication tend to have greater self-efficacy in managing and preventing the condition [93,94].

##### Digital Tools

According to the World Health Organization (WHO), the use of digital technologies is rapidly expanding in both the public and private sectors of healthcare [95]. Among the objectives of digital health is the promotion and support of sustainable lifestyle behaviors that contribute to the effective prevention and management of various health conditions [26]. Additionally, these platforms aim to reduce the need for unnecessary in-person healthcare visits by providing timely health information and guidance [26]. The widespread adoption of mHealth in perinatal care enables healthcare providers to support pregnant women through various digital means, including mobile phones, text messaging, emails, applications, online health journals, and integrated digital networks [96,97]. Findings from this review indicate that mHealth and other digital resources appear to be beneficial in promoting treatment adherence and facilitating behavior change among women with GDM [26,52,66]. Similarly, results from a RCT showed that GDM patients assigned to smartphone-supported care displayed improved compliance with blood glucose monitoring, better glycemic outcomes, and a reduced need for insulin therapy [98]. Furthermore, meta-analyses reported that mHealth interventions positively influenced glycemic control and reduced adverse pregnancy outcomes in individuals with GDM, and contributed to a lower incidence of neonatal intensive care unit admissions [66,99,100]. In contrast, evidence on the effectiveness of mHealth in supporting dietary management for GDM remains inconsistent [26,101]. Research suggests that effective digital solutions for nutritional therapy in GDM should be user-friendly for diet tracking, adaptable to individual dietary needs and cultural preferences, and grounded in an evidence-based framework [26,101]. In addition, data from a meta-analysis indicate that mHealth initiatives often prioritize behavioral change and treatment adherence, but frequently fail to acknowledge the impact of mental health challenges on self-care [102]. On the other hand, a meta-analysis found that lifestyle therapies based on mHealth may help alleviate symptoms of depression and anxiety in women with GDM [103]. Similarly, other studies have reported inconsistent effects of mHealth on psychosocial well-being during pregnancy, and the wide variation in research designs, settings, and assessment tools makes it difficult to generalize findings [104]. Existing research suggests that mHealth and digital tools may be especially beneficial for pregnant women from socioeconomically disadvantaged backgrounds, those with pre-existing health conditions, or those managing complex perinatal issues [104,105]. Nevertheless, there is a need for more research to evaluate the long-term implications of mHealth strategies for both maternal and child health [66].

##### Barriers and Facilitators

According to findings from this review, the management of GDM can be hindered by several barriers, including the negative psychosocial consequences of the condition, challenges in adhering to dietary therapy, competing work and family responsibilities, financial hardship, low health literacy, and a lack of individualized care from healthcare professionals. Facilitating factors include culturally sensitive approaches that are tailored to a woman’s literacy and income level, family and social support, and motivation to prevent negative health outcomes for the baby. Similar findings have been reported in the literature where cultural beliefs, food practices, and familial obligations can substantially influence GDM management [31,87,106,107,108].

1. Self-Management Challenges

Evidence suggests that many women feel overwhelmed and anxious following a GDM diagnosis, as they must quickly adapt to intensive lifestyle changes [89,109,110,111,112]. These include multiple daily finger pricks for blood glucose monitoring, which can be painful for some women, along with dietary modifications, increased physical activity, regular healthcare appointments, and, when necessary, insulin therapy requiring self-injection, more frequent blood testing, and a deeper understanding of dietary management as well as hypoglycemia prevention and treatment [89,109,110,111,112]. Conversely, some women perceived a GDM diagnosis as a positive opportunity to improve their lifestyle and make beneficial changes during pregnancy [113]. In addition, qualitative research indicates that participants with GDM often believed dietary restrictions and exercise during pregnancy could be potentially harmful to the baby, particularly in cultures where eating for two and limiting physical activity during pregnancy is the norm [107,114]. Moreover, evidence suggests that a lower level of education and poor health literacy are linked to a diminished understanding of the severity of GDM and the importance of its self-management [115,116,117]. Additionally, social customs, such as feeling obliged to accept food offered by friends or family, can sometimes conflict with clinical recommendations [85,87,118,119]. For many women, food carries deep emotional, cultural and social significance that extends far beyond its nutritional value, as research shows it often serves as a powerful cultural symbol that strengthens family bonds through social gatherings centered around traditional dishes, making dietary changes especially challenging [90,114,120]. In addition, women with GDM often report receiving conflicting or culturally insensitive nutritional advice from healthcare professionals, leading them to seek alternative information within their families and communities [65,90,107,114,118,121]. Studies revealed that to comply with GDM dietary advice, women frequently adapt their traditional recipes or cooking methods, and sometimes even prepare separate meals for themselves and their family, which quickly adds to their daily workload [113,118,120]. Additionally, evidence indicates that women’s perceptions of dietary advice are strongly shaped by the quality of their relationship with their healthcare providers [120]. Empathy, individualized care, and consistent follow-up build positive patient–provider relationships, while a lack of understanding leads some women to feel mistrust and seek information elsewhere [114,120].

2. Fatalistic Attitude

Research also indicates that fatalism, a belief that life events are supernaturally predetermined, which is prevalent in many cultures, has been identified as a determinant of GDM self-management [122]. This mindset can lead to a sense of powerlessness over health problems, making individuals feel that their condition cannot be changed [122]. Moreover, in cultures and communities with high diabetes prevalence, the widespread acceptance and normalization of diabetes and GDM can lead to fatalistic views, where diabetes development is seen as predetermined and unavoidable [89,117,123,124]. This outlook is linked to reduced perceived control over diabetic conditions and decreased adherence to recommended self-care routines, as people believe their actions have minimal impact on outcomes, which limits their self-efficacy and expectations for positive results [89,117,123,124]. To address these barriers, researchers advocate involving family members, friends, and community networks in diabetes education to counteract fatalistic beliefs and support both GDM management and T2D prevention [123].

3. Financial Hardship

Furthermore, qualitative evidence suggests that women with GDM from low socioeconomic backgrounds often face financial hardship and food insecurity, limiting their access to nutritious foods such as fresh fruits and vegetables [90,115]. As a result, they may rely on inexpensive energy-dense alternatives, particularly when their primary concern is ensuring their children do not go hungry [89,90]. Women with variable work schedules, irregular eating habits, and insufficient social support for childcare face challenges in maintaining consistent routines necessary for effective glycemic control [89,90]. These factors create substantial barriers to both blood glucose monitoring and treatment adherence [89,90]. The social and economic burdens of living in deprived areas can also intersect with family conflict, violence, unstable housing, and trauma, making lifestyle changes even more difficult while contributing to psychosocial stress that ultimately limits the time, energy, and resources needed to manage the condition effectively [90,115].

4. Disordered Eating

Additionally, stress, prenatal cravings, depression, and complicated relationship with food often contribute to emotional eating, such as indulging in comfort foods [89,90,125]. For women with a history of disordered eating, managing GDM can exacerbate dysregulated eating behaviors and lead to unhealthy choices [89,90,112,125,126]. Qualitative findings highlight that some women engage in overeating or binging on energy-dense foods, self-induced vomiting to control weight gain and glycemia, or overly restrictive eating to regulate blood glucose levels [89,90,112,125,126].

5. Culturally Appropriate Care

The literature indicates that important facilitators of GDM management include one-on-one culturally sensitive education tailored to individual needs, ideally delivered by healthcare professionals from similar cultural backgrounds who speak the patient’s preferred language, or with assistance of a trained professional interpreter [111,121,127,128,129]. In contrast, a one-size-fits-all approach is usually viewed as ineffective, reinforcing the need to involve healthcare providers and communities in tailoring culturally appropriate care, including support for sustained lifestyle changes postpartum to reduce the long-term risk of T2D [118,127,130].

#### 4.1.2. Interventions for GDM Prevention

##### Individual-Level Approaches

In terms of individual interventions to prevent the onset of GDM, this review found conflicting evidence from systematic reviews and meta-analysis regarding the most effective lifestyle strategies to adopt. The heterogeneity of results might be explained by the need for interventions to be compared within similar contexts and targeted groups, considering both individual and structural factors of influence. Although no single strategy has demonstrated universal efficacy, a multifaceted, personalized and culturally relevant approach may provide the most benefit in preventing GDM. Overall, the evidence from this review suggests that individualized interventions, particularly those initiated preconception or in early pregnancy, offer the greatest potential for reducing the risk of GDM [35,51]. Strategies that incorporate dietary modification and structured physical activity appear sometimes effective, especially when tailored to high-risk populations [34,39,51,54,58,59,62]. In contrast, recent research suggests that focusing exclusively on high-risk groups may unintentionally contribute to social stigma, potentially undermining individuals’ efforts to prevent the condition, whereas structural interventions that address the social and environmental determinants of GDM and T2D may represent a safer preventive strategy [131].

1. Diet

Findings from meta-analyses indicate that dietary patterns resembling the Mediterranean diet or the Dietary Approaches to Stop Hypertension (DASH) diet, when adopted before or early in pregnancy, are associated with a reduced risk of GDM [132,133,134,135,136,137,138]. The DASH diet, initially developed for hypertension management, promotes the consumption of fruits, vegetables, legumes, nuts, and moderate amounts of low-fat dairy, while limiting excess sodium, animal protein, and sweets [132,139,140]. Beyond blood pressure control, it has demonstrated benefits for cardiovascular health, metabolic syndrome, and glycemic control [141,142,143,144,145,146,147,148]. Similarly, the Mediterranean diet is rich in plant-based, fiber-dense foods and is associated with reduced chronic disease burden [149,150,151,152,153]. Although its precise definition varies by region, the traditional Mediterranean diet is generally characterized by high consumption of fruits, vegetables, legumes, nuts, whole grains, and olive oil, with moderate intake of fish and wine, and minimal amounts of red or processed meat [136,151,152,153,154]. The emphasis on complex carbohydrates and the resulting lower glycemic index may also mediate their protective effects against GDM [155,156]. High intake of dietary fiber and phytochemicals from fruits and vegetables have been linked to improved insulin sensitivity and reduced risk of T2D [157,158,159,160,161]. In contrast, diets high in animal protein, saturated fats, refined sugars, and low in fiber are associated with inflammation, obesity, insulin resistance, and increased cardiovascular risk [162,163,164,165,166,167,168,169,170,171,172]. A growing body of evidence indicates that oxidative stress and pathologic level of inflammation play a central role in pregnancy complications, including GDM, preeclampsia, and poor fetal outcomes [173,174,175,176,177,178,179,180,181,182,183,184]. The Mediterranean, DASH, and similar diets may help prevent GDM by modulating low-grade inflammation, according to research findings [170,185,186,187,188,189,190,191,192]. Obesity is also associated with disrupted inflammatory responses in maternal and fetal tissues and is an important risk factor for GDM [193,194,195,196,197,198,199]. Foods rich in antioxidants such as olive oil, fruits, and vegetables have been shown to reduce inflammatory markers [154,200,201,202]. Polyphenols and flavonoids found in olive oil and other plant-based foods may lower inflammation by downregulating pro-inflammatory gene expression [203,204,205,206,207]. Thus, dietary choices play a crucial role in modulating oxidative stress and inflammation, which is linked to the prevention of GDM [177,178,179,180,183,184,198,202]. It is worth noting that although moderate alcohol intake is traditionally part of the Mediterranean diet, its inclusion conflicts with pregnancy guidelines, as alcohol poses teratogenic and other health risks, with no known safe threshold at any stage [208,209,210]. Consequently, complete abstinence from alcohol is now recommended in many countries and should be clearly emphasized by healthcare providers advising women who are pregnant, breastfeeding, or planning to conceive [211,212,213,214,215,216].

2. Nutritional Supplements

Other findings from this review indicate that supplements of probiotics, myo-inositol, and vitamin D may be helpful to reduce the likelihood of developing GDM, although more clinical research is needed [51,54,62].

*Probiotics*

Findings from meta-analyses indicate that probiotic supplementation may lower the risk of developing GDM, enhance insulin sensitivity, and support glycemic control during pregnancy, while also potentially improving neonatal outcomes in women with GDM [217,218,219,220,221,222,223,224,225,226,227,228]. Emerging evidence further suggests that probiotics may be particularly effective in helping to prevent GDM when introduced early in pregnancy and administered over an extended duration [222,225,229]. These benefits are thought to result from favorable alterations in the gut microbiota, which modulate metabolic and inflammatory pathways, regulate lipid metabolism, and reduce oxidative stress [217,221,227,228,230]. However, the precise mechanisms through which probiotics influence insulin resistance and GDM risk remain unclear [231]. Moreover, substantial heterogeneity across studies contributes to inconsistent results. Further research is needed to determine the most effective strains, dosages, timing, and intervention models of the use of probiotics for the prevention and management of GDM.

*Myo-Inositol*

Myo-inositol is a nutriment essential for the formation of cell membranes and for mediating cellular responses to environmental stimuli [232]. It is an isomer of inositol, one of the intracellular mediators involved in insulin signaling, and is recognized as an insulin-sensitizing agent that supports glucose homeostasis [232,233,234,235,236]. According to findings from meta-analyses, myo-inositol supplementation may lower the incidence of GDM and its related complications [232,234,236,237,238,239,240,241,242,243,244,245]. While the beneficial effects of myo-inositol supplementation in the context of GDM appear promising, further investigation is needed to determine the optimal timing for initiation and appropriate dosage and intake frequency.

*Vitamin D*

Vitamin D deficiency appears to be linked to an increased risk of GDM, according to meta-analytic evidence, and supplementation may help support glycemic control, improve blood lipid profiles, and reduce adverse neonatal outcomes in individuals with GDM [246,247,248,249,250,251,252,253,254,255,256]. Nonetheless, the underlying biological processes remain to be clearly defined through additional studies.

3. Physical Activity

Evidence suggests that physical activity interventions are generally safe and beneficial for women with GDM and should be encouraged in the absence of obstetric contraindications [4]. The American College of Obstetricians and Gynecologists (ACOG) recommends that pregnant women with diabetes receive individualized exercise prescriptions to ensure safety and effectiveness [257]. Women using insulin or other glucose-lowering agents need to be informed of the risk of hypoglycemia, as medication doses may require adjustments based on physical activity levels [4,258]. Dietitians with expertise in prenatal care and GDM play a key role in aligning dietary recommendations with exercise regimens and insulin therapy [4,258]. A growing body of evidence from systematic reviews and meta-analyses indicates that supervised, low-to-moderate-intensity exercise initiated in the first trimester can reduce the incidence of GDM, particularly among women who are not overweight or obese [132,138,259,260,261,262]. In contrast, the effectiveness of physical activity alone in women with overweight or obesity remains inconclusive. While some studies highlight a potential benefit when adherence to exercise is high, others report limited or no effect on GDM risk in this group [263,264]. Nonetheless, early implementation of individualized lifestyle interventions focusing on realistic goal setting, a low-glycemic/hypocaloric diet, and regular moderate-intensity exercise, has been associated with reduced GWG and GDM risk in women with elevated BMI [265,266,267,268]. According to findings from a cohort study of 14,451 women, the likelihood of developing GDM increased consistently with higher pre-pregnancy BMI [269]. Compared to women with normal weight, those who were overweight or obese prior to pregnancy had nearly twice and more than twice the odds of developing GDM, with odds ratios of 1.91 and 2.55, respectively [269]. In pregnancies complicated by GDM, both pre-pregnancy obesity and excessive GWG are contributors to complications such as fetal growth disturbances, hypertensive disorders, congenital malformations, macrosomia, stillbirth, and an increased likelihood of cesarean delivery [269,270,271,272,273,274,275,276,277]. These risks extend beyond pregnancy, with associations noted between maternal GDM and postpartum weight retention as well as increased BMI in offspring during childhood and adulthood [278,279,280,281,282,283,284,285,286]. Research also highlights that any participation in physical activity before or during early pregnancy has been associated with a 21–46% reduction in the odds of developing GDM, compared to no activity at all [34,136,287]. Beyond GDM prevention, prenatal exercise is linked to additional maternal and neonatal health benefits, such as modest reductions in maternal weight gain, and significantly lower risks of adverse outcomes including macrosomia, preterm birth, cesarean delivery, fetal growth restriction, and birth trauma in women with GDM [288,289,290]. Maternal benefits also include improved fitness and enhanced psychological well-being [291]. GDM shares many pathophysiological features with T2D, such as insulin resistance, systemic inflammation, and increased adiposity [284,287,292]. Physical activity may counteract these mechanisms by reducing body fat and enhancing insulin sensitivity [287,293,294]. Furthermore, it promotes glucose uptake in skeletal muscle through upregulation of glucose transporter type 4 (GLUT4) and glycogen synthase activity [293,294,295]. Moreover, exercise-induced secretion of interleukin-6 (IL-6) from muscle cells has anti-inflammatory effects by inhibiting cytokines like tumor necrosis factor alpha (TNF-α) and interleukin-1 beta (IL-1β), contributing to reduced insulin resistance [294,296,297,298].

Evidence suggests that women diagnosed with GDM are often motivated to adopt healthier lifestyle behaviors during pregnancy, viewing the diagnosis as a catalyst to protect their unborn child and improve their health habits [113,299,300]. Nutrition has been recognized as the cornerstone of effective diabetes prevention and management during pregnancy, and the literature highlights the importance of personalized nutrition counseling in the context of GDM [4,258,301,302,303,304,305]. However, although the benefits of physical activity during pregnancy are well documented, many women encounter barriers, including time constraints, fatigue, nausea, physical discomfort, and uncertainty about exercise safety [113,299,300]. To address these issues, exercise prescriptions should integrate culturally adapted behavioral strategies and take into account key social and environmental factors, such as access to safe places to exercise, family responsibilities, employment conditions, income level, and social support networks, that influence physical activity behaviors [113,299,300,306,307]. Although staying active before and during pregnancy offers clear benefits, research indicates that it is usually not sufficient on its own to control GWG and manage GDM effectively [4]. Thus, despite promising evidence, high heterogeneity across studies makes it challenging to interpret the true impact of exercise in the context of GDM prevention. This variability may be attributed to differences in study design, physical activity measurement methods, and intervention characteristics, including the type of exercise (e.g., aerobic activities like walking, cycling, or swimming; resistance training including weightlifting and pelvic floor exercises), as well as the timing, frequency, intensity, supervision, and setting of exercise sessions. Other methodological concerns include poor participant compliance, unmeasured confounding factors such as diet, and inconsistent control of these variables across studies. Consequently, further high-quality research is needed to clarify the characteristics of optimal exercise interventions for GDM, how these can be tailored to individual and cultural contexts, and their true effectiveness when accounting for potential confounding factors.

##### Population-Level Approaches

1. Environmental Factors

Research indicates that city policies and environmental planning can help prevent obesity and chronic diseases [308,309,310,311,312]. Community layout is recognized as an important determinant of population health as it can influence dietary choices, physical activity, and leisure time by ensuring, for example, walking distance to facilities and shops, as well as the availability of safe bicycle paths [309,310,311,312,313]. Evidence suggests that the design of buildings and cities might also influence people’s social integration and isolation, impacting their mental health [309]. Research shows that environmental influences can benefit body and mind, including access to green spaces, and having safe and friendly streets [309,314]. Living in greener neighborhoods has been associated with better mental health, with specific improvements observed in symptoms of anxiety and depression [315]. This may be due to the mental restoration provided by natural settings and their role in facilitating social contact and community engagement [315]. A prospective cohort study in the UK (N = 23,865) with a mean follow-up of 11.3 years found that individuals living in the greenest neighborhoods had a 19% lower relative risk of developing T2D compared to those in the least green environments; this association remained statistically significant after adjusting for age, sex, BMI, parental diabetes, and socioeconomic status [310]. Similarly, data from a systematic review and meta-analysis indicated that while urban residence was associated with a higher risk and prevalence of T2D, walkable environments and access to green space were consistently linked to reduced risk and prevalence [311]. Findings from a Danish cohort study (N = 42,775) reported that both commuter and recreational cycling were associated with a reduced risk of T2D [313]. Additionally, cross-sectional research revealed that individuals living in more walkable neighborhoods engaged in higher levels of physical activity, drove less, and exhibited slightly lower obesity rates, regardless of demographic factors [312].

According to findings from this review, exposure to green space during pregnancy supports healthier maternal glucose regulation, whereas limited access is linked to higher risks of GDM and related complications such as preeclampsia, preterm birth, and depression. Multiple cohort studies consistently show that greater proximity to green environments corresponds with lower blood glucose markers in pregnant women, while those in areas with sparse green space, poor neighborhood walkability, or food deserts (areas with limited access to affordable, healthy food options), face elevated odds of metabolic and mental health challenges [37,42,50,56]. Similar findings have been reported in the literature supporting the benefits of green space and walkability on GDM risk and glycemic control. A prospective cohort found that pregnant individuals with pregestational diabetes residing in highly walkable neighborhoods achieved better glycemic control in both early and late pregnancy [316]. Similarly, a retrospective cohort indicated that greater residential green space exposure during the second trimester was significantly linked to a lower risk of developing GDM [317]. Data from a randomized, cluster-controlled trial further revealed that the protective effect of urban greenness on GDM risk was especially strong among socioeconomically disadvantaged women, underscoring the heightened value of green spaces for vulnerable groups [318]. Nevertheless, neighborhoods with lower socioeconomic status frequently have limited green space availability, and residents often worry about the safety and quality of nearby parks and recreational facilities [56]. Ensuring more equitable distribution, access, and safety to green space could serve as a strategy to address health disparities linked to economic disadvantage in underserved communities. Therefore, thoughtful urban design and community planning can promote well-being both before and during pregnancy while offering the advantage of not requiring individual behavioral modifications, ultimately leading to sustained public health benefits. This perspective has been increasingly embraced at national levels, including by Canada’s Chief Public Health Officer, as part of a broader initiative to prevent disease by fostering healthier living environments [314].

2. Health in All Policies

Finally, findings from this review highlight the usefulness of multisector collaboration in enhancing population health and addressing persistent societal challenges, such as perinatal health inequities. These disparities stem from complex interconnections between medical and social determinants, necessitating coordinated efforts among local authorities and professionals across the medical, social, and public health sectors [33]. The WHO defines HiAP as an approach that acknowledges the profound influence of non-health sectors on population well-being, emphasizing that health outcomes are largely shaped by social and economic conditions governed outside the healthcare system [319]. By integrating health considerations into policymaking at all levels, national, regional, and local, HiAP shifts the focus from individual responsibility to systemic and policy-related drivers of health [320]. This approach aims to implement policies that impact areas such as public safety, housing, transportation, agriculture, education, and marketing, to promote health equity and prevent diseases [319,321,322,323,324]. Accordingly, evidence shows that environmental and socioeconomic determinants of health have a substantial impact on the incidence, morbidity, and mortality rates of chronic diseases, including diabetes and perinatal health outcomes [33,37,131,325,326,327,328]. As explained by WHO, social determinants of health are non-medical factors that influence well-being, encompassing the conditions in which people are born, grow, live, work, and age, shaped by the distribution of money, power, and resources [329]. Key determinants include income and social status, education and literacy, employment and working conditions, social support networks, culture, race and ethnicity, gender, and access to healthcare and healthy environments [329,330,331]. A focus on HiAP requires cooperation with the healthcare system and all stakeholders to reverse the growing prevalence of GDM and its consequences [33,332]. By targeting upstream determinants of health, HiAP promotes more aligned and effective policymaking, linking policies from various sectors to health outcomes [320]. Improving health, therefore, involves, for example, reducing unemployment and social exclusion, ensuring income security, improving housing, enhancing education, and implementing policies on the marketing of unhealthy foods and beverages [320]. As outlined in Frieden’s framework for public health action [333], preventive interventions that target the broader determinants of health typically have the greatest population-level impact while requiring the least individual effort, compared to downstream strategies that focus on individual behavior change after a disease diagnosis, such as GDM [333]. This underscores the importance of addressing the underlying causes of GDM to halt its increasing prevalence and associated outcomes. However, despite the recognized value of intersectoral collaboration in advancing HiAP, research indicates that implementation often remains challenging due to weak institutional frameworks, unclear accountability across sectors, and limited financial resources [334,335]. Evidence also highlights that such collaboration is frequently hindered by structural, cultural, and practical barriers; however it can be overcome through strong political backing, a shared vision, and the ability to align interests across sectors [33,60,332,334,335]. Insights from Finland’s long-standing HiAP initiative demonstrate that this framework can effectively advance public health in contemporary societies [336,337]. Its success in Finland has relied on sustained intersectoral collaboration, which requires health sector personnel to have the time, expertise, and resources to engage with other sectors, along with access to comprehensive data on health and its determinants, and the capacity to analyze policy impacts across all levels of governance [336,337]. Broad public support also depends on ensuring that the general population, political leaders, civil servants, and the media understand health literacy concepts and their wider societal implications [336,337]. In addition, legislative foundations have proven especially valuable for maintaining the long-term consistency and sustainability of HiAP initiatives [336,337].

### 4.2. Recommendations for Practice, Policy, and Research

To improve the management of GDM, healthcare providers should adopt holistic, person-centered approaches that consider each individual’s unique circumstances, including socioeconomic status, cultural background, and health literacy level, and how these factors influence adherence to treatment. Recommendations for nutrition and physical activity need to be tailored to reflect considerations like affordability, accessibility, cultural and religious preferences, and family responsibilities. Psychological support should also be offered from the beginning of pregnancy and continue through the postpartum period to help prevent and manage emotional distress and depression. Additionally, integrating mHealth digital tools into standard care may help enhance women’s ability to self-manage their condition. Postpartum services need to be offered to support women in sustaining positive long-term lifestyle changes to prevent T2D.

Preventing GDM requires moving beyond individual-level interventions to address the upstream social and environmental determinants of health. Preventive strategies should include investment in preconception care, especially for underserved populations, to address disparities and improve maternal and neonatal health outcomes. Policies must acknowledge that GDM risk is shaped by broader conditions like poverty, neighborhood safety, food security, access to green spaces, housing quality, and job stability. This means rethinking policy priorities and investing in initiatives such as healthy food programs, safe and walkable infrastructure, equitable employment practices, and affordable housing; factors that collectively create environments supportive of metabolic and reproductive health. To achieve this, policy frameworks like HiAP can guide intersectoral strategies to ensure health considerations are integrated across sectors like transportation, housing, urban planning, labor, and education.

Further research is essential to address knowledge gaps in the prevention and management of GDM. There is a growing need to study effective training models for healthcare providers, including strategies for interprofessional collaboration in the culturally competent care of patients with GDM, both before and after professional licensure. Well-designed trials are also needed to evaluate the effectiveness of non-pharmacological interventions, including physical activity, diet, and supplementation. Special attention should be given to high-risk groups such as migrants and individuals with low socioeconomic status, to identify effective, culturally appropriate strategies. Furthermore, research should investigate the role of GDM-related stigma, its prevalence across various populations including ethnic minorities, and its impact on mental health, glycemic control, and overall maternal and fetal health. Understanding how social determinants of health, especially those linked to racial, ethnic, and socioeconomic inequalities, affect outcomes in women with GDM should be research priority. Finally, studies must assess the impact of broader policy changes on maternal health and identify how best to adapt interventions for diverse communities.

### 4.3. Strengths and Limitations

This integrative review has both strengths and limitations. Among its strengths are the inclusion of numerous peer-reviewed studies, many of which are systematic reviews and meta-analyses, and the inclusion of varied methodological approaches allowing for a more comprehensive and multifaceted examination of the research topic. All included studies were assessed to be of medium to high methodological quality. Nevertheless, synthesizing findings from multiple study designs can be complex and potentially introduce bias. This was mitigated through a structured, rigorous approach based on established frameworks. However, the review only included literature published in English, which may have excluded relevant studies in other languages. Furthermore, as most included articles originated from high-income countries, the generalizability of the findings to developing nations may be limited.

## 5. Conclusions

The rising prevalence of GDM, with its profound impact on maternal and child health and substantial burden on healthcare systems, underscores the need for a comprehensive, multi-level public health response. This integrative review highlights that effective GDM management requires interventions that are culturally appropriate, adapted to individual literacy levels, and incorporate psychological and social support. Digital health tools also offer a valuable avenue for improving patient adherence.

Prevention efforts should focus on pre-pregnancy lifestyle modifications, including plant-rich diets and regular physical activity, while some nutritional supplements appear promising but need further clinical validation. Importantly, addressing the root causes of GDM, including its environmental, socioeconomic, and racial determinants of health, is essential for mitigating disease incidence and severity. A multisectoral approach, such as the HiAP framework, can be helpful in fostering collaboration across sectors to build supportive environments, promote healthy behaviors, and reduce inequities and disease burden. Further research is needed to support effective preventive and management measures beyond standard pharmacological treatment, so that evidence-based solutions can be applied to enhance and safeguard the health of current and future generations.

## Figures and Tables

**Table 1:** Characteristics of the studies included from the literature search.

#	Author/s, Year	Country	Design	Study Population	Main Findings of Relevance	MMAT Score
1	Adesina et al. (2021) [26]	UK	Systematic review	Pregnant women with GDM or history of GDM (16 included studies; N = 2593)	The adoption of digital tools may be an effective approach to GDM self-management.	4/5
2	Bennett et al. (2018) [27]	Australia	Systematic review and meta-analysis	Pregnant women (45 included studies; N = 15,293)	Diet and PA interventions designed to reduce GWG were more effective than standard care in reducing the incidence of GDM.	4/5
3	Breuing et al. (2020) [28]	Germany	Scoping review	People at risk of GDM or T2D (125 included studies)	Barriers in the prevention of GDM/T2D included limited knowledge, social support, and economic factors.	4/5
4	Byrn & Penckofer (2015) [29]	U.S.	Cross-sectional study. Q.	Pregnant women (N = 135)	Women with GDM were 3.79 times more likely to have a history of depression (95% CI [1.07, 13.45], *p* = 0.04) than women without GDM after controlling for age, income, marital status, BMI, and gravida.	4/5
5	Carolan (2014) [30]	Australia	Qualitative study. I.	Diabetes educators (N = 6)	A culturally and literacy appropriate approach is needed for disadvantaged women with GDM.	5/5
6	Carolan et al. (2012) [31]	Australia	Qualitative study. FG., I.	Pregnant women with GDM (N = 15)	Women from migrant and low socioeconomic backgrounds often struggled to comprehend and adhere to GDM dietary and PA guidelines.	5/5
7	Carolan-Olah et al. (2017) [32]	U.S.	Systematic review	Hispanic pregnant women with GDM or history of GDM (7 included studies; N = 1887)	Intensive nutritional counseling, low GI diet, and culturally tailored interventions may help with GMD management.	5/5
8	Daalderop et al. (2024) [33]	Netherlands	Mixed methods. I., Q.	Professionals (Interviews: N = 81, Questionnaires: N = 85)	In the context of perinatal health, structural, cultural, and practical obstacles hinder cross-sectoral interactions. To find bottlenecks in cross-sectoral collaboration, it is helpful to examine facilitators and barriers at these three levels.	4/5
9	Davenport et al. (2018) [34]	Canada	Systematic review and meta-analysis	Pregnant women without contraindication to exercise (106 included studies; N = 273,182)	Findings demonstrated lower odds of developing GDM with exercise-only interventions compared with no exercise. Interventions combining exercise and cointerventions were less effective than exercise alone for GDM.	5/5
10	Donazar-Ezcurra et al. (2017) [35]	Australia	Systematic review	Pregnant women (35 included studies)	Some dietary patterns, such as the Mediterranean diet, seem to lower the risk of developing GDM.	4/5
11	Elsenbruch et al. (2007) [36]	Germany	Cross-sectional study. Q.	Pregnant women (N = 896)	Low social support throughout pregnancy was associated with higher rates of depressive symptoms and a lower quality of life.	5/5
12	Field et al. (2024) [37]	U.S.	Prospective cohort study. SDA.	Pregnant women (N = 9155)	After adjusting for known covariates, pregnant women residing in food deserts or in neighborhoods with low walkability exhibited higher odds of developing GDM.	4/5
13	Guo et al. (2023) [38]	China	RCT	Pregnant women and their partners (N = 140)	The Couples Coping with GDM Program was associated with improvements in GDM knowledge and self-management.	5/5
14	Guo et al. (2019) [39]	China	Meta-analysis	Pregnant women with GDM (47 included studies; N = 15,745)	Exercise of moderate intensity for 50–60 min twice a week could lead to an approximately 24% reduction in GDM.	4/5
15	Haron et al. (2023) [40]	Malaysia	Systematic review	Pregnant women with GDM (19 included studies; N = 2237)	Self-care education for women with GDM had a positive impact on GDM outcomes.	5/5
16	Hasani et al. (2020) [41]	Iran	Cross-sectional study. Q.	Pregnant women with GDM (N = 212)	Improving women’s social capital could enhance their GDM self-management.	4/5
17	He et al. (2025) [42]	China	Birth cohort study	Pregnant women with GDM (N = 5814)	Higher residential greenness exposure corresponded to reduced HbA1c levels between mid-pregnancy and late pregnancy in women diagnosed with GDM.	5/5
18	Helm et al. (2022) [43]	U.S.	Scoping review	Pregnant women with GDM (12 included studies; N = 2193)	Findings highlight the efficacy of technology-supported diabetes self-management education and medical nutrition therapy.	4/5
19	Huang et al. (2022) [44]	U.S.	Systematic review	Women with GDM or at risk of GDM (7 included studies; N = 2569)	Enhanced, culturally tailored care for GDM is essential, given that immigrant women in the U.S. experience higher rates of GDM than their U.S.-born counterparts.	4/5
20	Jung et al. (2021) [45]	Korea	Systematic review	Pregnant women with GDM or a history of GDM (14 included studies)	Psychosocial supportive interventions can positively affect self-care behaviors, lifestyle changes, and physiological parameters in women with GDM.	5/5
21	Karavasileiadou et al. (2022) [46]	Saudi Arabia	Systematic review	Pregnant women with GDM (10 included studies)	Findings highlight overlooked aspects of GDM including a lack of individualized care and a lack of options regarding follow-ups with healthcare professionals. Some women felt abandoned after giving birth.	4/5
22	Khatri et al. (2023) [47]	Australia	Scoping review	Multisectoral actions (40 included studies)	Multisectoral actions support primary healthcare by promoting cross-sector policies and engaging stakeholders across all levels of the health system.	4/5
23	Lagisetty et al. (2017) [48]	U.S.	Systematic review	Adults (34 included studies)	Improving risk factors for the development of diabetes in ethnic minority populations can be achieved by culturally specific interventions.	5/5
24	Laredo-Aguilera et al. (2020) [49]	Spain	Systematic review	Pregnant women with GDM (7 included studies; N = 782)	Any type of PA of sufficient intensity and duration can have benefits for pregnant women with GDM.	4/5
25	Liao et al. (2019) [50]	China	Prospective birth cohort study	Pregnant women (N = 6807)	Residing in areas with more green space was significantly linked to lower maternal blood glucose levels and a reduced risk of developing impaired glucose tolerance and GDM.	4/5
26	Lim et al. (2023) [51]	U.S.	Systematic review and meta-analysis	Women of childbearing age (116 included studies; N = 40,940)	Lifestyle interventions, and myo-inositol/inositol reduced the risk of GDM.	5/5
27	McGovern et al. (2024) [52]	Ireland	Qualitative meta-synthesis	Pregnant women with GDM or obesity (29 included studies; N = 604)	Women highlighted the importance of self-monitoring, information trustworthiness, peer support, motivational tools, and convenience in achieving behavior change using mHealth technology.	5/5
28	Oostdam et al. (2011) [53]	Netherlands	Systematic review and meta-analysis	Pregnant women (19 included studies)	Results indicate that there may be some benefits of dietary counseling, LGI diet advice, or an exercise program. No strong conclusions can be drawn about the best intervention for prevention of GDM.	4/5
29	Quotah et al. (2024) [54]	UK	Systematic review and meta-analysis	Women at risk of GDM (84 included studies; N = 22,568)	GDM risk was reduced using combined diet and PA, inositol and vitamin D supplementation in women identified in early pregnancy as higher risk.	4/5
30	Roesler et al. (2024) [55]	Australia	Cross-sectional study. S.	Women with GDM (N = 815)	Findings suggest a demand for more supportive, person-centered GDM care, improved information provision, and individualized implementation of clinical guidelines.	4/5
31	Runkle et al. (2022) [56]	U.S.	Retrospective birth cohort study	Pregnant women (N = 238,922)	Pregnant women in the lowest tertiles of per-person green space, walkable green space, and overall green space faced the highest risks of GDM and mental health disorders. Those with the least green space exposure had elevated risks of preeclampsia, preterm birth, and depression.	4/5
32	Russo et al. (2015) [57]	U.S.	Systematic review and meta-analysis	Pregnant women (10 included studies; N = 3401)	Results suggest that PA in pregnancy provides a slight protective effect against the development of GDM.	5/5
33	Sampathkumar et al. (2023) [58]	UK	Systematic review and meta-analysis	Pregnant women (30 included studies; N = 257,876)	A strong link was found between higher self-reported physical activity and healthy diets during the pre-pregnancy period and reduced risk of GDM.	4/5
34	Takele et al. (2024) [59]	Australia	Systematic review and meta-analysis	Pregnant women with GDM (116 included studies; N = 40,940)	Dietary, PA, diet plus PA, and myo-inositol interventions reduced the incidence of GDM.	5/5
35	Tancred et al. (2024) [60]	UK	Scoping review	Multisectoral activities (93 included documents)	The pathway to HIAP includes robust coordination and leadership, governance and policymaking and implementation capacities, intersectoral/multisectoral strategies, information systems, and transparent, resource-financed and investment opportunities.	4/5
36	Tanentsapf et al. (2011) [61]	Danmark	Systematic review	Pregnant women (13 included studies; N = 2486)	Dietary advice during pregnancy appeared effective in decreasing total GWG and long-term postpartum weight retention.	5/5
37	Tang et al. (2022) [62]	China	Systematic review and meta-analysis	Pregnant women (46 included studies; N = 16,545)	PA and probiotic intervention were more effective than placebo in reducing the risk of developing GDM.	4/5
38	Thangaratinam et al. (2012) [63]	UK	Meta-analysis of RCT	Pregnant women (44 included studies; N = 7278)	Dietary and lifestyle interventions during pregnancy can reduce maternal GWG and improve outcomes for both mother and baby. Interventions based on diet were the most effective and associated with reductions in maternal GWG and improved obstetric outcomes.	5/5
39	Wah et al. (2019) [64]	Australia	Qualitative study	Migrant women of Chinese ethnicity pregnant with GDM (N = 18)	Women described difficulty meeting their dietary needs when they conflicted with family interest.	5/5
40	Wan et al. (2019) [65]	Australia	Systematic review and meta-analysis	Ethnic Chinese women with GDM (29 included studies; N = 3944)	LGI diets and fiber-enriched diets were associated with improved glycemic control and pregnancy outcomes.	5/5
41	Wei et al. (2023) [66]	China	Systematic review and meta-analysis	Pregnant women with GDM (27 included studies; N = 3483)	Compared with standard care, Internet-based mHealth interventions were more effective in controlling BGL and improving maternal and infant outcomes in patients with GDM.	4/5
42	Wu et al. (2022) [67]	China	Systematic review and meta-analysis	Overweight/obese pregnant women (23 included studies; N = 8877)	Diet intervention alone or combined diet + PA intervention can be considered viable strategies for overweight or obese pregnant women to restrict GWG.	5/5
43	Zeinabeh et al. (2023) [68]	Iran	Cross-sectional study. Q.	Pregnant women with GDM (N = 78)	Stress and BGL can be reduced by early intervention and provision of mindfulness counseling in women under treatment with GDM diet, especially in the first half of pregnancy.	4/5
44	Zhang et al. (2021) [69]	China	Cross-sectional study. SDA.	Pregnant women with GDM (N = 106)	Individualized PA prescription plus dietary management can help women with GDM in the second-and-third trimester to control their BGL and BMI value within a healthy range.	4/5

Note. BGL = blood glucose levels; BMI = body mass index; CI = confidence interval; DM = diabetes mellitus; FG = focus group(s); GDM = gestational diabetes mellitus; GI = glycemic index; GWG = gestational weight gain; Hb = hemoglobin; HIAP = health in all policies; I = interview(s); LGI = low glycemic index; MMAT = mixed methods appraisal tool; N = sample size; PA = physical activity; Q = questionnaire(s); RCT = randomized controlled trials; S = survey(s); SDA = secondary data analysis; T2D = type 2 diabetes; UK = United Kingdom; U.S. = United States.

## Data Availability

Not applicable.

## Abbreviations

ACOG	American College of Obstetricians and Gynecologists
BMI	Body mass index
DASH	Dietary Approaches to Stop Hypertension
GDM	Gestational Diabetes Mellitus
GLUT	Glucose transporter
GWG	Gestational weight gain
Hb	Hemoglobin
HiAP	Health in All Policies
IL	Interleukin
LGI	Low-glycemic index
MeSH	Medical Subject Headings
MMAT	Mixed Methods Appraisal Tool
mHealth	Mobile health
PCOS	Polycystic ovary syndrome
RCT	Randomized controlled trial
TNF-α	Tumor necrosis factor alpha
T2D	Type 2 diabetes
UK	United Kingdom
U.S.	United States
WHO	World Health Organization
